# Supplementary figures and images for: Variation in virion phosphatidylserine content drives differential GAS6 binding among closely related flaviviruses
Source: J Virol. 2025 Sep 24;99(10):e01111-25. doi: 10.1128/jvi.01111-25 (PMC12548462; doi:10.1128/jvi.01111-25)

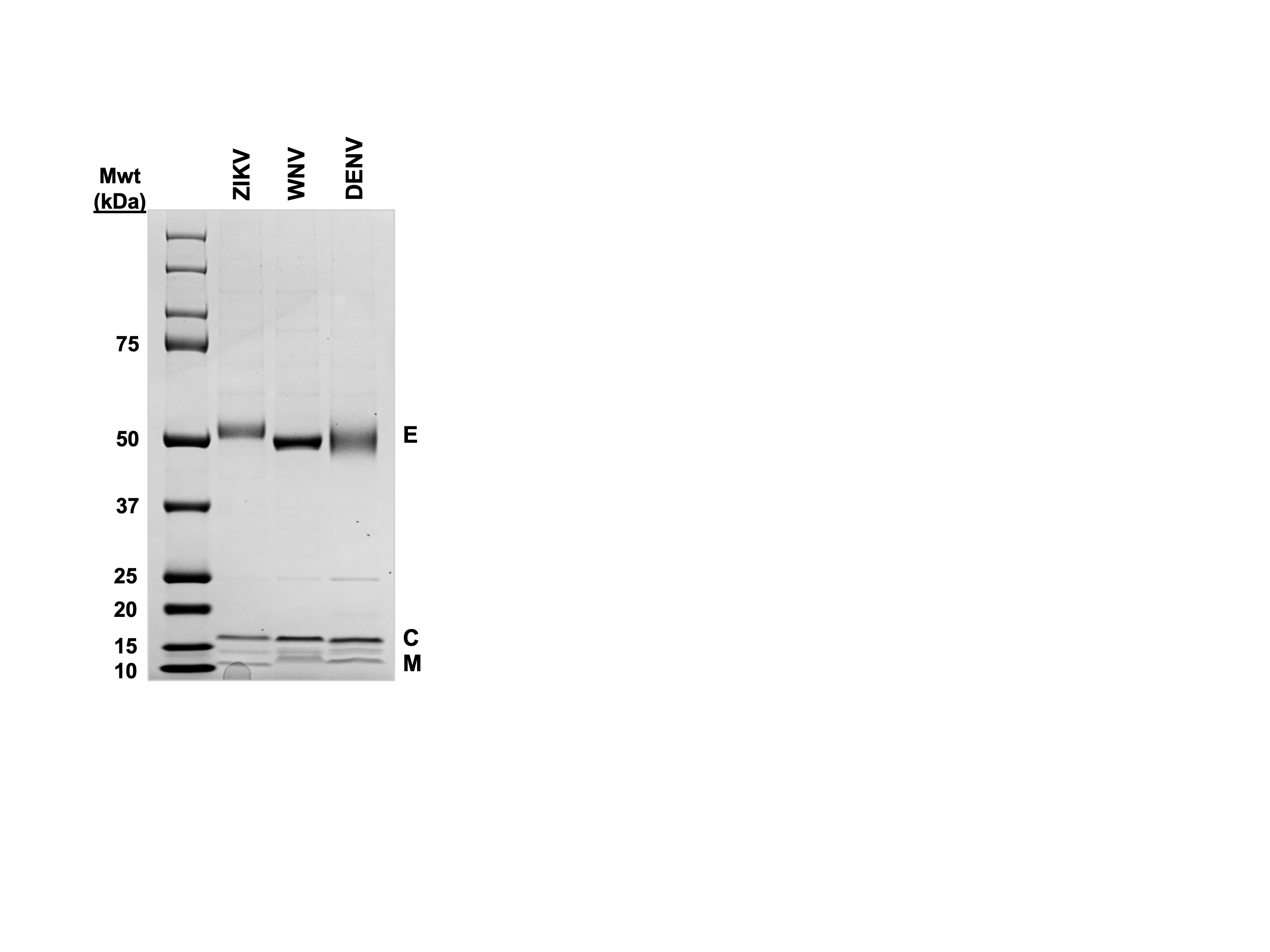

Supplement: Figure S1 — Purified ZIKV, WNV, and DENV used for lipid analysis. [file jvi.01111-25-s0001.tiff]

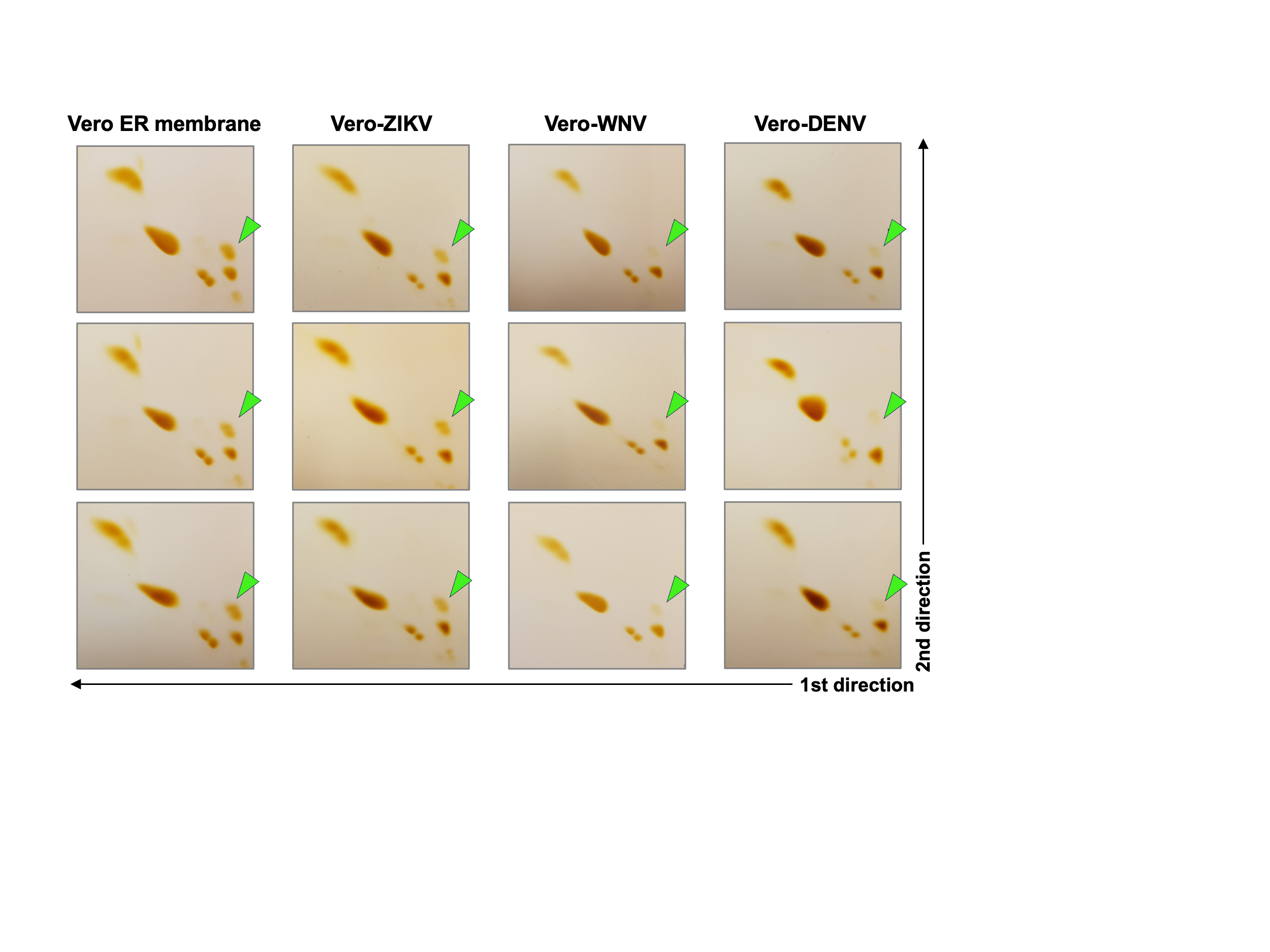

Supplement: Figure S2 — PS content of ZIKV is substantially higher than that of WNV and DENV. [file jvi.01111-25-s0002.tiff]

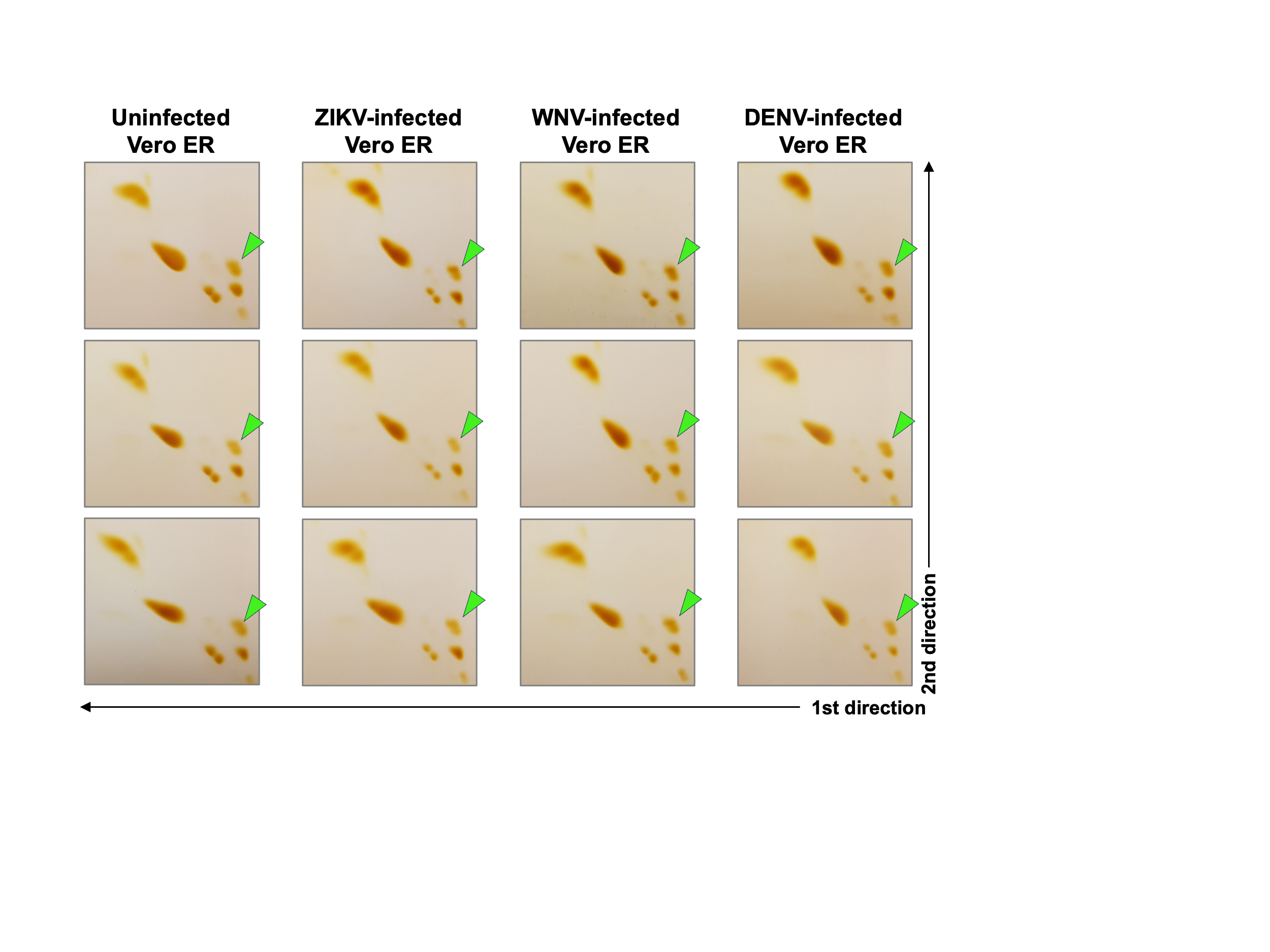

Supplement: Figure S3 — PL composition of the ER membrane is not differentially altered by different viruses. [file jvi.01111-25-s0003.tiff]
