# Supplementary material for: Variation in virion phosphatidylserine content drives differential GAS6 binding among closely related flaviviruses
Source: J Virol. 2025 Sep 24;99(10):e01111-25. doi: 10.1128/jvi.01111-25 (PMC12548462; doi:10.1128/jvi.01111-25)
Supplement: Supplemental legends — Legends for Fig. S1 to S3. [file jvi.01111-25-s0004.docx]

**SUPPLEMENTAL FIGURE LEGENDS**

**Figure S1. Purified ZIKV, WNV, and DENV used for lipid analysis.** Purity of the viruses were grown in Vero cells and purified through potassium tartrate gradient centrifugation was examined by SDS-PAGE and Coomassie staining prior to lipid extraction. 2.5 x 10^10^ genome copies of each virus were loaded.

**Figure S2. PS content of ZIKV is substantially higher than that of WNV and DENV.** Total lipids were extracted from the isolated ER membrane of Vero cells or from the viruses grown in Vero cells and purified by potassium tartrate centrifugation. The extracted lipids were separated by 2D TLC, and phospholipid spots were visualized by iodine vapor. Green arrowheads point PS spots. Each plate represents an independent experiment conducted with an independently prepared sample.

**Figure S3. PL composition of the ER membrane is not differentially altered by different viruses.** Vero cells were uninfected or infected with the indicated virus at an MOI of 1 and harvested two (ZIKV and WNV infected cells) or six (DENV infected cells) days post infection. The ER membranes were isolated, total lipids were extracted from them, lipids were separated by 2D TLC, and phospholipid spots were visualized by iodine vapor. Green arrowheads point PS spots. Each plate represents an independent experiment conducted with an independently prepared sample.
